# Sodium Content in Cereal-Based Products Sold in Italy: How Far Are We from the Global Benchmarks?

**DOI:** 10.3390/nu14153088

**Published:** 2022-07-27

**Authors:** Daniela Martini, Pasquale Strazzullo, Mauro Serafini, Marisa Porrini, Nicoletta Pellegrini, Donato Angelino

**Affiliations:** 1 Department of Food, Environmental and Nutritional Sciences (DeFENS), Università degli Studi di Milano, 20133 Milan, Italy; daniela.martini@unimi.it (D.M.); marisa.porrini@unimi.it (M.P.); 2 Department of Clinical Medicine and Surgery, Federico II University of Naples Medical School, 80131 Naples, Italy; 3 Faculty of Bioscience and Technology for Food, Agriculture and Environment, University of Teramo, 64100 Teramo, Italy; mserafini@unite.it (M.S.); dangelino@unite.it (D.A.); 4 Department of Agricultural, Food, Environmental and Animal Sciences, University of Udine, 33100 Udine, Italy

**Keywords:** cereal-based products, salt, sodium reduction, sodium targets, reformulation

## Abstract

Reformulation of food products is one of the measures needed for reducing salt consumption. Accordingly, the World Health Organization (WHO) recently proposed global sodium benchmarks for different food categories to be used for setting national policies. Therefore, the sodium content of cereal-based products currently sold in Italy was compared with the WHO benchmarks, highlighting those categories primarily needing a reformulation. To this aim, the sodium content and several declarations (i.e., nutrition and health claims, organic or gluten free declaration) were retrieved from 2917 cereal-based products sold on the Italian market. All “minimally processed breakfast cereals” had a sodium content below the benchmark, while “flatbreads” and “leavened bread” had the highest percentage of items above the respective sodium benchmarks. Flatbreads and “crackers/savory biscuits” showed the highest median delta values from the respective benchmarks of 360 and 278 mg/100 g, respectively. Large variability in terms of percentage of products with sodium content above the benchmark was observed within the same categories, as well as among products with different declarations. A large number of food products currently sold on the Italian market have a sodium content above the benchmark. This result suggests the need to reformulate many food products currently on the market to achieve the WHO/United Nations (UN) objective of 30% global reduction in sodium intake by 2025.

## 1. Introduction

The European Food Safety Authority (EFSA), in agreement with the World Health Organization (WHO) [1], declared that data retrieved from studies on the relationships between sodium intake and health outcomes do not allow enabling an average requirement or population reference intake for sodium [2]. However, both health agencies set a safe and adequate intake of sodium for the general adult population (>16 years) of 2 g/day, corresponding to 5 g/day of salt (sodium chloride) [1,2]. Moreover, the WHO set a goal of 30% relative reduction in the mean population intake of salt/sodium by 2025 as a global target within the Global Action Plan for the Prevention and Control of Noncommunicable Diseases 2013–2020 [3]. Actually, sodium intake in a large part of the general adult European population remains higher than the WHO recommended level, particularly in Eastern and Southern European countries [4,5]. A cross-sectional survey of almost 2000 Italians, aged 35–74 years, found an age-standardized mean daily population salt intake of 10.8 g in men and 8.3 g in women in 2008–2012, based on 24-h urinary sodium excretion. Despite a statistically significant reduction in salt intake observed in 2018–2019, its intake result was still far higher than the limit of 5 g/day [6].

A large body of literature shows that excessive salt intake is associated with high blood pressure [7], which in turn drives an increased risk of stroke and cardiovascular disease [8].

Based on the findings of the Global Burden Disease (GBD) study, in 2017, the high intake of sodium (>3 g/die) was responsible for three million deaths and 70 million disability-adjusted life-years (DALYs) [9]. Despite the GBD observed an appreciable decrease of global age-standardized rates of death and DALY attributable to high sodium intake from 1990 to 2019, the total numbers of deaths and DALYs attributable to high sodium intake increased due to population growth and aging, especially in developed countries [10]. It was recently estimated that achieving the WHO target of 5 g of salt per day per adult by 2030 would avoid 87,870 cases of premature ischemic heart diseases, and 126,010 premature strokes by 2050 in the UK [11]. This is mainly attributable to the linear decrease in blood pressure following the reduction in salt intake [12], as well as to the preservation of normal kidney function [13].

In recent years, a series of Salt Reduction Initiatives Around the World were put in place to push the WHO 2025 Global Salt Reduction Target [3,14]. These approaches include, among others, interventions such as consumer education, front-of-pack labeling, and salt taxation, which have been all implemented in many countries in recent years. A crucial component of the population strategy for addressing this target involves the commitment by the food industry to reformulate food products to progressively reduce their sodium content. In fact, salt is present in only tiny amounts in almost all natural foods and it is added to reach much higher concentrations by the food industry for various reasons, including to improve the palatability of foods and to extend shelf-life.

In this scenario, the WHO recently proposed a series of global sodium benchmarks for different food categories [15] that should be useful for countries in setting national policies as well as for fostering the dialogue between health institutions and the private sector at the global level. Among the 18 categories of foods considered, specific benchmarks were established for cereal-based products, which include products such as bread and bakery products, which are known to be among the major contributors to dietary salt intake in most populations (25–40% of the total daily intake) [16]. In Italy, cereal-based products, and among them mostly breads, are the major sources of dietary salt [17].

Based on these premises, the aims of the present work were to: (i) compare the sodium content of cereal-based products currently sold in Italy with the WHO benchmarks; (ii) establish the gap between the current sodium content of these products and the respective benchmarks; (iii) assess which are the main subcategories and types that need reformulation in the next years. This work was conceived within the Food Labeling of Italian Products (FLIP) study, which systematically investigates the nutritional quality of food products currently sold in Italy.

## 2. Materials and Methods

### 2.1. Food Product Selection

The cereal-based product selection from the major retailers present on the Italian market offering a home-shopping section in their website has been deeply described elsewhere [18,19,20].

For each cereal-based item present in the FLIP database, the following information was retrieved: company name, brand name, descriptive name, salt content (g/100 g), presence or absence of nutrition claims (NC), health claims (HC), gluten free (GF) declaration, organic declaration (presence or absence of organic declaration) and being branded or private label products. Based on the descriptive name and according to WHO [15], cereal-based products were classified in nine subcategories: (i) “Cookies/sweet biscuits”; (ii) “Cakes and sponges”; (iii) “Pies and pastries”; (iv) “Crackers/savory biscuits”; (v) “Minimally processed breakfast cereals”; (vi) “Highly processed breakfast cereals”; (vii) “Sweet and raisin breads”; (viii) “Leavened bread”; (ix) “Flatbreads”. Other subcategories and types of cereal-based products (e.g., cereal bars, raw pasta) considered in the FLIP study were not included since no sodium benchmarks have been established for these subcategories yet. Salt content was used to calculate sodium content (as salt divided by 2.5) and in turn the delta of sodium content from the relative benchmark for each item was calculated.

### 2.2. Statistical Analysis

Statistical analysis was carried out using IBM SPSS Statistics^®^ (Version 27.0, IBM corp., Chicago, IL, USA) and performed at *p* < 0.05 of significance level. The normality of data distribution was preliminarily verified through the Kolmogorov–Smirnov test and rejected. Therefore, variables were expressed as median and interquartile range (IQR). The Mann–Whitney non-parametric test for two independent samples was applied to evaluate the difference between the sodium/salt content (g/100 g product) of each product from the related global benchmark.

## 3. Results

### 3.1. Number and Types of Included Products

The number of collected products is reported in Table 1. A total of 2917 products was retrieved, with most of the items being “crackers/savory biscuits” (*n* = 808), followed by “cookies/sweet biscuits” (*n* = 753) and “leavened breads” (*n* = 337), while the lowest number of items was retrieved for “minimally processed breakfast cereals” (*n* = 49). When focusing on the salt/sodium content, a large variability was observed both across and within the considered cereal-based food subcategories. Subcategories highest in sodium were “crackers/savory biscuits” (median 720 (IQR 480–900) mg/100 g), “flatbreads” (666 (600–800) mg/100 g) and “leavened breads” (508 (452–560) mg/100 g), while “minimally processed breakfast cereals” were those with the lowest sodium content (4 (1–12) mg/100 g).

### 3.2. Comparison of Sodium Content in Food Subcategories and Benchmarks

The extrapolated sodium amount of each food subcategory was compared with the related sodium benchmarks (Table 1). “Minimally processed breakfast cereals” was the only subcategory where all the items had a sodium content below the benchmark (100 mg/100 g). “Sweet and raisin breads”, “cookies/sweet biscuits” and “highly processed breakfast cereals” subcategories had 15%, 34% and 45%, respectively, of the items with the sodium content above the benchmarks (set at 310, 265 and 280 mg/100 g, respectively). Among other subcategories, “flatbreads” and “leavened bread” had the highest number of items (98% and 92% of products) above the related sodium benchmarks (set at 320 and 330 mg/100 g, respectively).

Within food subcategories, the percentage of products with salt content above the benchmark also varied substantially. For instance, among “crackers/savory biscuits”, the percentage of items above the benchmark ranged from 6% for “rice and corn cakes” to 93% for “taralli”, while among “cookies/sweet biscuits” from 1% for “cream-filled wafer” to 41% for “short-bread biscuits” (Table 1).

Table 2 reports the distribution of items with sodium content equal or below and above the global benchmarks, according to other characteristics (i.e., brand, organic, GF declaration, presence of NC or HC). Comparing branded and private label products, a higher percentage of branded products from “cake and sponges” and “leavened bread” subcategories had a sodium content equal or below the benchmark, while GF “cookies/sweet biscuits” and “crackers/savory biscuits” had more often a lower Na content compared to their gluten-containing counterparts. Finally, for both the presence of NC and the organic declaration, a large variability was observed among subcategories since an almost equal number of those with and without these declarations had a sodium content equal or below the benchmarks.

**Figure 1:**
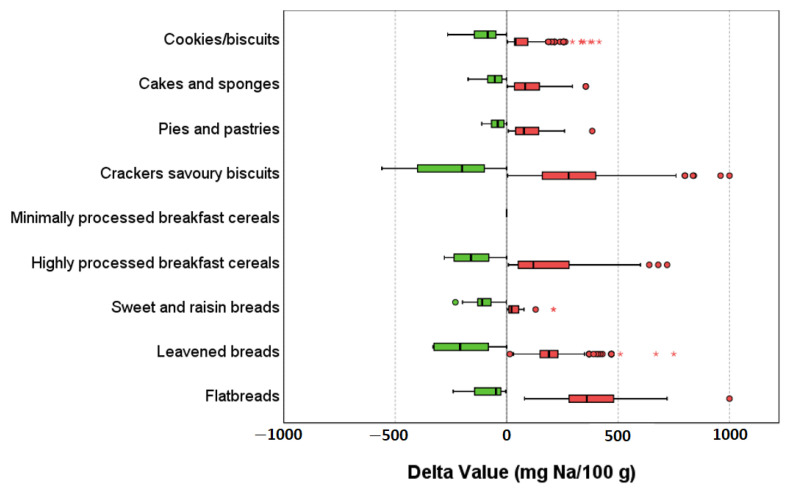
Box-plot representation of the delta sodium (Na) content from the related benchmarks in the different subcategories of cereal-based products. Legend: green and red bars represent the delta values for products with sodium content equal or below and above the benchmark, respectively. Dots and asterisks represent mild and extreme outliers, respectively.

Figure 1 graphically shows the median delta values between the sodium content of the different products and the related benchmark set per subcategory by WHO [15], while Figure S1 in Supplementary Materials shows the same analysis, but detailed for each type of product.

Regarding products with the sodium content above the benchmarks, the subcategories with the greatest differences from the target were “flatbreads” and “crackers/savory biscuits” (360 (280–480) mg/100 g and 278 (160–400) mg/100 g, respectively) (Figure 1). Within this last subcategory, the types particularly differing from the benchmarks for their delta sodium values were “taralli” (320 (280–414) mg/100 g) and “croutons, bruschetta and “frisella” bread” (280 (120–480) mg/100 g) (Supplementary Materials Figure S1). Considering products with a sodium content equal or below the benchmarks, leavened breads were characterized by the lowest median delta value (−209 (−326–−270) mg/100 g, followed by “crackers/savory biscuits” with (−200 (−400–−100) mg/100 g). Among the types, “rice and corn cakes” (−400 (−480–−200) mg/100 g), “loaf bread” (−325 (−326–−141) mg/100 g) and “breadsticks” (−208 (−260–−200) mg/100 g) were the products with the lowest median delta values from benchmark targets (Supplementary Materials Figure S1).

## 4. Discussion

By comparing the sodium content of cereal-based products currently sold on the Italian market with the respective benchmarks proposed by the WHO [15], we found most of the items have a sodium content much higher than the related benchmarks, especially among “flatbreads”, “leavened breads” and “crackers/savory biscuits”. Although the benchmarks set for these subcategories are relatively high, the sodium content and the respective delta values of the products included in these food groups are remarkably high, ranging from 190 mg for leavened breads to 360 mg for flat breads, corresponding to about 0.5–0.9 g of salt, respectively. This represents a serious challenge, particularly for bread, which in Italy is the main category of products contributing to the daily salt intake [17]. Based on the guidelines suggested consumption of 3.5 servings of bread/day (of 50 g each) [21], the very high salt content of leavened bread (190 mg/100 g over the WHO benchmark) leads to an excess sodium intake of 285 mg/day (corresponding to 0.7 g/day of salt). Therefore, this finding calls for urgent action of reformulating at least this bread subcategory.

Another important finding was that for most subcategories and food types thereof a large variability in sodium content is seen with several products equal or below and as many others above the respective benchmark. A clear example is represented by the “crackers/savory biscuits” subcategory, which includes both types with low (e.g., “rice and corn cakes”) and high percentages (e.g., “taralli”) of products with sodium content above the benchmark. This finding highlights the pivotal importance of consumers’ education in reading the nutrition facts in order to buy foods with low salt content especially in those food items commonly used as bread substitutes. Large variability was also observed among products with different declarations, such as NC, organic or GF declaration, suggesting that the presence of these declarations on the food pack cannot be a reliable proxy of a generally lower sodium content.

A thorough comparison of these findings with previous studies conducted in Italy is not possible since, to the best of our knowledge, this is the first study comparing the sodium content of food products currently on the Italian market with the benchmarks set by the WHO, which have been published very recently [15]. A similar survey has been performed on 7234 packaged foods available in Canada on 2010–2011, finding that 48.6% of products exceeded the sodium benchmark levels set by Health Canada [22]. Among the different food categories, 36.3% of breakfast cereals and 46.8% of bakery products exceeded the benchmark, mainly breadcrumbs and croutons (58.9%) and packaged bread products (50.1%). In another survey performed in 14 Latin American and Caribbean countries, 18% of the 16,000 packaged foods exceeded regional targets (generally equal or higher than those set by the WHO), among which 36% of cakes, 17% of bread products, 9% of breakfast cereals and 3% of flavored cookies and crackers [23]. Interestingly, despite the high number of products in line with the sodium targets, population’s sodium intake in the same countries far exceeds recommendations, thus suggesting a large consumption of discretionary salt and likely the need for more stringent sodium targets to effectively reduce sodium intake.

Other studies reported the changes that occurred in the sodium content of foods over time in countries with various salt reduction policies. Pombo-Rodrigues and coworkers analyzed several different breakfast cereal products sold in the UK from 2004 to 2015 and detected a significant 47% reduction in their salt content which was related to the UK salt reduction program implemented in 2004 [24].

A similar survey was conducted on food products sold in the Netherlands from 2011 to 2016 [25]. A significant reduction was observed in this time-frame in the average salt content of bread, but not of bread substitutes or breakfast cereals. Pravst et al. investigated changes in the sodium content of prepacked foods sold in Slovenia during 2011–2015 [26]. The authors found an increased sodium content in cakes, muffins, and pastry and no significant changes for prepacked plain bread, with sodium values still ~530 mg/100 g, and thus are quite far from the global WHO benchmarks. Taken together, these results suggest that further efforts are needed to address salt reduction in foods in different markets worldwide. Despite this, it is noteworthy that a wide range of initiatives has been already put in place at national and international levels [14], and also in Italy [27]. Most of the strategies are based on two parallel approaches: (i) nutrition education programs to consumers, to let them aware of the need to reduce their salt intake and, consequently, to modify their intention-to-buy; and (ii) improvement of the nutritional quality of food products through reformulations aimed at reducing their sodium content [14].

Regarding reformulation, a recent UK systematic review showed that among 20 studies focused on the impact of product reformulation to reduce sodium intake, 13 reported estimated reduced sodium intakes in consumers between 4% and 15% per year, with an average of −0.57 g/day [28]. The same authors performed a survey on more than 2000 UK citizens in the years 2008/2009 and 2016/2017 reporting a −16% salt intake, mainly driven by reformulation and renewal of food products, thus supporting the importance of these initiatives [29]. Conversely, a recent survey performed in Australia calculated the impact of reformulation of packaged foods to meet the targets set by the Australian government through the Healthy Food Partnership [30]. The authors estimated that also assuming a complete compliance to the target, this would lead to a small reduction in the sodium content of purchased products (50 mg/day) not significantly impacting on sodium intake [30]. Intriguingly, however, when they simulated a reformulation using the UK targets, they showed a predicted double reduction of the salt intake, close to 110 mg/day, suggesting the need to set more stringent targets to achieve significant results.

According to a very recent systematic review [14], 68 countries worldwide are currently working with the food industry for food reformulation and of these, 57 countries have established salt targets. Among these, 19 countries have set mandatory maximum salt limits: in about half of these countries, the mandatory targets have been set only for bread, whereas in the others they cover a wider range of foods, including processed meats, cheeses, crisps and snacks, soups and stocks, canned products, i.e., fish, tomato, fruit and vegetables. In all the other countries, the negotiation with industry led to the definition of voluntary salt targets, with a wide range in the number of food categories and type of products (within categories) being targeted for reformulation. Previous systematic reviews and modeling studies have suggested that mandatory/legislative approaches may be associated with larger reductions in population salt intake [31,32]. In this regard, the “Farm to Fork” strategy has planned to launch initiatives to stimulate reformulation of food, including the setting of maximum levels for certain nutrients by Q4 2021 [33].

While food reformulation guided by the definition of food targets is an obligatory tool in order to reduce salt intake at the population level, it has been objected that the change in the salt content of the products may affect consumers’ acceptance. Indeed, a meta-analysis of nine studies investigating the effect of 10–92% salt reduction in bread [34] concluded that salt could be reduced by approximately 40% without significantly impacting consumer acceptability, thus supporting food companies in making deep reductions in salt when reformulating food products. It is intriguing to notice that with this reduction, all bread items considered in the studies included in the meta-analysis would have a sodium content below the WHO benchmark.

The present study has several strengths and some limitations worth to be highlighted. First, in this survey, we have investigated the sodium content of almost 3000 items, which is a high number representative of products currently on the market belonging to food subcategories that are among the main contributors of salt intake in the Italian population as well as of energy and other important nutrients [17]. One of the limitations is, of course, that these data do not cover other food subcategories, such as cured meats, cheese, and canned products, that should be surveyed in the future for their contribution to salt intake. Moreover, in the present study, we investigated the sodium content of all cereal-based products currently sold in selected retailers, but excluded other channels such as discount stores or vending machines. Furthermore, we just considered the salt content of the products currently on the market, regardless their effective selling rate, which is obviously relevant to influence the real salt intake of the Italian population. In this regard, an evaluation adjusted by considering the sales volumes of the different items would allow establishing priorities for food reformulation in order to have the greatest impact on the reduction of sodium intake in Italy.

## 5. Conclusions

The present study pointed out that cereal-based products, among the most relevant contributors of salt intake in the Italian population, show a wide variability in salt content and, in most of the products, have median values above the sodium benchmarks of WHO. This finding supports and boosts the need for urgent negotiations between public health authorities, government representatives and food companies, with the contribution of experts from the scientific societies, to define a strategy for reformulation of cereal-based foods and likewise other food subcategories based on the definition of specific targets according to WHO indications. In addition to this action, appropriate surveys at regular time points in the future, together with the ones investigating the Italian dietary habits, will be necessary to monitor food reformulation and to investigate the potential impact of sodium reduction on the health of the Italian population.

## Figures and Tables

**Table 1:** Number of cereal-based products, in terms of subcategories and types, their salt and sodium content, the relative sodium benchmarks and the percentage of the items above the benchmark.

					Items with Sodium Content above the Benchmark	
Subcategories and Types	Total Number of Items	Salt Content (g/100 g) *	Sodium Content (mg Na/100 g) *	Benchmark (mg Na/100 g) **	*n*	%
**Cookies/sweet biscuits**	**753**	**0.54 (0.37–0.75)**	**220 (150–300)**	**265**	**254**	**34**
Tea cookies	254	0.60 (0.40–0.75)	240 (160–300)		93	37
Short-bread biscuits	346	0.61 (0.48–0.75)	246 (192–300)		142	41
Cream-filled wafer	77	0.31 (0.25–0.38)	124 (100–152)		1	1
Covered and/or sandwich cookies	76	0.48 (0.30–0.63)	190 (118–250)		18	24
**Cakes and sponges**	**280**	**0.53 (0.39–0.73)**	**209 (152–290)**	**205**	**143**	**51**
Cream-filled sponge cake	74	0.37 (0.30–0.50)	146 (120–200)		15	20
Yogurt plumcake and muffin	126	0.67 (0.51–0.82)	274 (204–336)		91	72
Sponge cake	74	0.51 (0.41–0.71)	200 (164–284)		35	47
Cream-filled and/or covered chilled snack	6	0.23 (0.19–0.55)	92 (78–219)		2	33
**Pies and pastries**	**107**	**0.36 (0.25–0.50)**	**150 (100–200)**	**120**	**64**	**60**
Cream/jam-filled pies	61	0.41 (0.32–0.55)	164 (128–220)		46	75
Italian traditional pastries	27	0.20 (0.13–0.30)	80 (52–120)		6	22
Other pastries	19	0.38 (0.18–0.64)	150 (72–256)		12	63
**Crackers/savory biscuits**	**808**	**1.80 (1.20–2.25)**	**720 (480–900)**	**600**	**490**	**61**
Crackers	183	1.90 (1.50–2.25)	760 (600–900)		135	74
Breadsticks	193	2.00 (1.80–2.30)	800 (720–920)		176	91
Rice and corn cakes	142	0.50 (0.30–1.00)	200 (120–400)		8	6
Taralli	99	2.30 (2.10–2.50)	920 (840–1000)		92	93
Croutons, bruschetta and “frisella” bread	96	1.80 (1.47–2.50)	720 (588–1000)		67	70
Rusks	95	1.25 (1.00–1.50)	500 (400–600)		12	13
**Minimally processed breakfast cereals**	**49**	**0.01 (0.00–0.03)**	**4 (1–12)**	**100**	**0**	**0**
Muesli	1	0.02 (0.02–0.02)	8 (8–8)		0	0
Flakes	19	0.01 (0.00–0.03)	4 (1–12)		0	0
Bran cereals	10	0.03 (0.00–0.07)	10 (0–28)		0	0
Puffed cereals	18	0.01 (0.00–0.01)	4 (0–4)		0	0
Other cereals	1	0.03 (0.03–0.03)	12 (12–12)		0	0
**Highly processed breakfast cereals**	**271**	**0.63 (0.30–1.00)**	**252 (120–400)**	**280**	**122**	**45**
Muesli	67	0.30 (0.11–0.53)	120 (44–212)		4	6
Flakes	107	0.90 (0.48–1.30)	360 (192–520)		77	72
Bran cereals	11	1.20 (0.59–1.30)	480 (236–520)		8	73
Puffed cereals	20	0.47 (0.02–1.00)	186 (6–400)		7	35
Other cereals	66	0.59 (0.38–0.79)	236 (152–316)		26	39
**Sweet and raisin breads**	**168**	**0.57 (0.46–0.73)**	**228 (184–292)**	**310**	**25**	**15**
**Leavened bread**	**337**	**1.27 (1.13–1.40)**	**508 (452–560)**	**330**	**310**	**92**
Loaf bread	95	1.25 (0.93–1.50)	500 (372–600)		76	80
Rolls	70	1.30 (1.20–1.30)	520 (480–520)		69	99
Sliced bread	172	1.25 (1.17–1.40)	500 (468–560)		165	96
**Flatbreads**	**144**	**1.67 (1.50–2.00)**	**666 (600–800)**	**320**	**141**	**98**

* Values are expressed as median and interquartile range. ** Reference: [15]. Bold means subcategories.

**Table 2:** Number and percentages of the cereal-based products with sodium content equal or below and above to the relative benchmarks, grouped for the presence of other declarations on the food pack.

Subcategories	Branded *n* (%)	Private Label *n* (%)	Organic *n* (%)	Conventional *n* (%)	Gluten Free *n* (%)	Gluten Containing *n* (%)	with NC *n* (%)	without NC *n* (%)	with HC *n* (%)	without HC *n* (%)
**Cookies/sweet biscuits**										
Na above the benchmark	158 (34)	96 (33)	23 (26)	231 (35)	11 (22)	243 (35)	103 (49)	151 (28)	1 (13)	253 (24)
Na equal or below the benchmark	307 (66)	192 (67)	66 (74)	433 (65)	38 (78)	461 (65)	109 (51)	390 (72)	7 (87)	492 (76)
**Cakes and sponges**										
Na above the benchmark	67 (41)	77 (66)	14 (64)	130 (50)	17 (55)	127 (51)	28 (60)	116 (50)	0 (0)	144 (52)
Na equal or below the benchmark	98 (59)	39 (34)	8 (36)	129 (50)	14 (45)	123 (49)	19 (40)	118 (50)	2 (100)	135 (48)
**Pies and pastries**										
Na above the benchmark	32 (59)	32 (60)	6 (86)	58 (58)	2 (100)	62 (59)	7 (78)	57 (58)	0 (0)	64 (60)
Na equal or below the benchmark	22 (41)	21 (40)	1 (14)	42 (42)	0 (0)	43 (41)	2 (22)	41 (42)	0 (0)	43 (40)
**Crackers/savory biscuits**										
Na above the benchmark	290 (59)	200 (63)	48 (25)	442 (71)	25 (18)	465 (70)	121 (42)	369 (71)	23 (58)	467 (61)
Na equal or below the benchmark	200 (41)	118 (37)	141 (75)	177 (29)	114 (82)	204 (30)	169 (58)	149 (29)	17 (42)	301 (39)
**Minimally processed** **breakfast cereals**										
Na above the benchmark	-	-	-	-	-	-	-	-	-	-
Na equal or below the benchmark	28 (100)	21 (100)	36 (100)	13 (100)	1 (100)	48 (100)	33 (100)	16 (100)	8 (100)	41 (100)
**Highly processed** **breakfast cereals**										
Na above the benchmark	54 (46)	68 (44)	25 (40)	97 (46)	9 (64)	113 (44)	91 (47)	31 (39)	27 (60)	95 (42)
Na equal or below the benchmark	64 (54)	85 (56)	37 (60)	112 (54)	5 (36)	144 (56)	101 (53)	48 (61)	18 (40)	131 (58)
**Sweet and raisin breads**										
Na above the benchmark	10 (12)	15 (18)	3 (60)	22 (13)	0 (0)	25 (15)	1 (4)	24 (17)	0 (0)	25 (15)
Na equal or below the benchmark	74 (88)	69 (82)	2 (40)	141 (87)	1 (100)	142 (85)	27 (86)	116 (83)	2 (100)	141 (85)
**Leavened bread**										
Na above the benchmark	34 (79)	276 (94)	176 (90)	134 (95)	30 (91)	280 (92)	75 (81)	235 (96)	2 (100)	308 (92)
Na equal or below the benchmark	9 (21)	18 (6)	20 (10)	7 (5)	3 (9)	24 (8)	18 (19)	9 (4)	0 (0)	27 (8)
**Flatbreads**										
Na above the benchmark	32 (94)	109 (99)	95 (97)	46 (100)	5 (100)	136 (98)	12 (86)	129 (99)	2 (100)	139 (98)
Na equal or below the benchmark	2 (6)	1 (1)	3 (3)	0 (0)	0 (0)	3 (2)	2 (14)	1 (1)	0 (0)	3 (2)

NC, nutrition claim; HC, health claim.

## Data Availability

The data presented in this study are available on request from the corresponding author.

## Supplementary Materials

The following supporting information can be downloaded at: https://www.mdpi.com/article/10.3390/nu14153088/s1, Figure S1: Box-plot representation of the delta sodium content from the related benchmarks in the different types of cereal-based products. Legend: HP: highly processed; MP: minimally processed.

## Appendix A

**Table A1:** SINU Young Working Group.

Margherita Dall’Asta	Department of Animal Science, Food and Nutrition, Università Cattolica del Sacro Cuore, Piacenza, Italy
Gaetana Paolella	Department of Chemistry and Biology “A. Zambelli”, University of Salerno, Fisciano, Italy
Marika Dello Russo	Institute of Food Sciences, National Research Council, Avellino, Italy
Stefania Moccia,	Institute of Food Sciences, National Research Council, Avellino, Italy
Carmela Spagnuolo	Institute of Food Sciences, National Research Council, Avellino, Italy
Daniele Nucci	Veneto Institute of Oncology IOV-IRCCS, Padova, Italy
Veronica Pignone	Freelance Nutritionist, San Giorgio del Sannio, Italy
Emilia Ruggiero	Department of Epidemiology and Prevention, IRCCS Neuromed, Pozzilli, Italy
Alice Rosi	Department of Food and Drug, University of Parma, Italy
Giorgia Vici	School of Biosciences and Veterinary Medicine, University of Camerino, Italy
