# Supplementary material for: Sodium Content in Cereal-Based Products Sold in Italy: How Far Are We from the Global Benchmarks?
Source: Nutrients. 2022 Jul 27;14(15):3088. doi: 10.3390/nu14153088 (PMC9370200; doi:10.3390/nu14153088)
Supplement: Supplementary file 1 [file nutrients-14-03088-s001.zip › nutrients-1804746-supplementary.pdf]

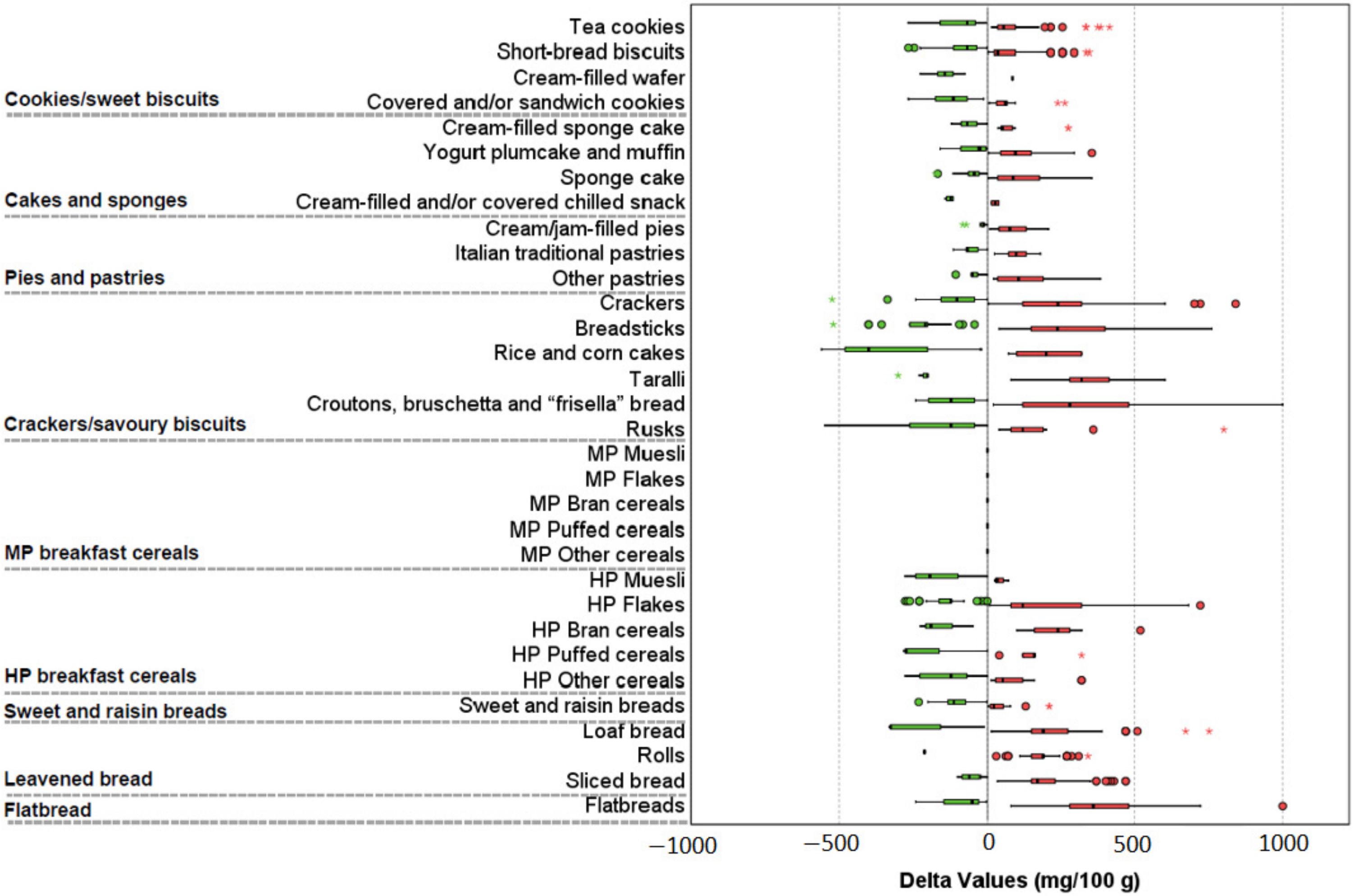

Figure S1. Box-plot representation of the delta sodium content from the related benchmarks in the different types of cereal-based products. Legend: HP: highly processed; MP: minimally-processed. Dots and asterisks represent mild and extreme outliers, respectively.
